# A Striking Coronary Artery Pattern in a Grown-Up Congenital Heart Disease Patient

**DOI:** 10.1155/2016/5482578

**Published:** 2016-01-06

**Authors:** Fortunato Iacovelli, Martino Pepe, Gaetano Contegiacomo, Vito Alberotanza, Filippo Masi, Alessandro Santo Bortone, Stefano Favale

**Affiliations:** ^1^Interventional Cardiology Service, “Montevergine” Clinic, Via Mario Malzoni, 83013 Mercogliano, Italy; ^2^Division of Cardiology, Department of Advanced Biomedical Sciences, University of Naples “Federico II”, Via Sergio Pansini 5, 80131 Naples, Italy; ^3^Section of Cardiovascular Diseases, Department of Emergency and Organ Transplantation, University of Bari “Aldo Moro”, Piazza Giulio Cesare 11, 70124 Bari, Italy; ^4^Interventional Cardiology Service, “Santa Maria” Clinic, Via Antonio De Ferrariis 22, 70124 Bari, Italy; ^5^Section of Diagnostic Imaging, Interdisciplinary Department of Medicine, University of Bari “Aldo Moro”, Piazza Giulio Cesare 11, 70124 Bari, Italy; ^6^Section of Heart Surgery, Department of Emergency and Organ Transplantation, University of Bari “Aldo Moro”, Piazza Giulio Cesare 11, 70124 Bari, Italy

## Abstract

Left ventricular noncompaction (LVNC) is a myocardial disorder probably due to the arrest of normal embryogenesis of the left ventricle. It could be isolated or associated with other extracardiac and cardiac abnormalities, including coronary artery anomalies. Despite the continuous improvement of imaging resolution quality, this cardiomyopathy still remains frequently misdiagnosed, especially if associated with other heart diseases. We report a case of LVNC association with both malposition of the great arteries and a very original coronary artery pattern.

## 1. Introduction

Left ventricular noncompaction (LVNC) is a myocardial disorder, which is thought to occur due to the arrest of normal embryogenesis of the left ventricle (LV), leading to distinct morphological characteristics in the ventricular chamber [1]. The affected segments had a two-layer structure: a compact epicardial layer and an endocardial layer consisting of a prominent trabecular meshwork and deep intertrabecular spaces [2]; these features are found predominantly in the apical and the mid ventricular segments of the LV. It is classified in isolated NC and in ventricular NC associated with other extracardiac and cardiac abnormalities, including coronary artery anomalies [3]. The prevalence varies considerably among different series and is still unknown; several limitations for this assessment are the different diagnostic criteria, the heterogeneous populations, and the retrospective design of most studies [4]. Because of continuous improvement of imaging resolution quality, this cardiomyopathy is increasingly diagnosed, even if it remains frequently misdiagnosed especially in the cases of LVNC associated with other heart defects [5]. Clinical signs are variable, ranging from lack of symptoms to heart failure and thromboembolic events, arrhythmias till sudden cardiac death [6], also if the pathophysiologic mechanisms of these severe manifestations in LVNC are partially unclear.

We present herein a case of LVNC association with both malposition of the great arteries and a very original coronary pattern. This report has been approved by the Institutional Review Board, with informed consent given by the patient.

## 2. Case Presentation

A 30-year-old male, smoker, without family history for congenital or acquired cardiovascular diseases, was referred to our department because of chest/epigastric pain and syncope due to ventricular fibrillation after cocaine overdose. After the usual resuscitation maneuvers, including 3 early direct current shocks, orotracheal intubation, and pharmacological support, his clinical conditions were stabilized. The first echocardiographic evaluation highlighted hypokinesia of the inferior LV wall, with mild systolic dysfunction and mild-to-moderate mitral valve regurgitation. During hospitalization he progressively developed lesion, ischemia, and then necrosis waves in diaphragmatic leads at the electrocardiogram (ECG); moreover the toxicological test results confirmed the recent cocaine abuse, while laboratory ones showed increased troponin I (peak 23.9 ng/mL) and N-Terminal pro-Brain Natriuretic Peptide (peak 2356 pg/mL) levels.

**Figure 1:**
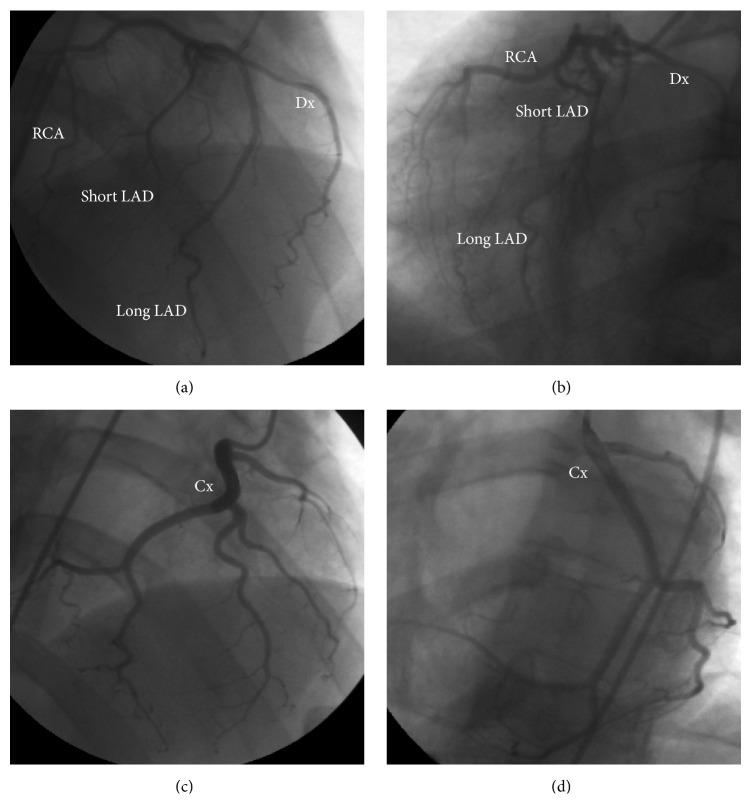
Coronary angiography (CRA 24 RAO 10 and CAU 24 LAO 34) showing dual LAD ((a), (b)) and (CRA 24 RAO 12 and CAU 21 LAO 32) lone Cx giving off the posterior descending artery ((c), (d)). CRA: cranial; RAO: right anterior oblique; CAU: caudal; LAO: left anterior oblique.

**Figure 2:**
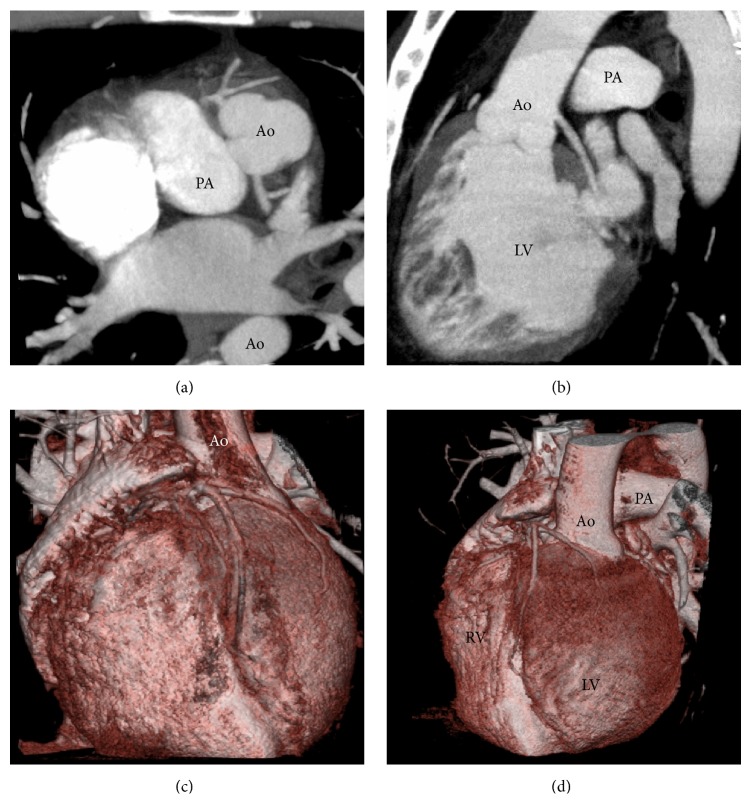
Paraxial (a) and parasagittal (b) maximum intensity projection reconstruction from cardiac CT scan, demonstrating the anomalous site of the coronary ostia as well as the malposition of the great arteries, confirmed by 3D volume-rendered reconstruction ((c), (d)).

**Figure 3:**
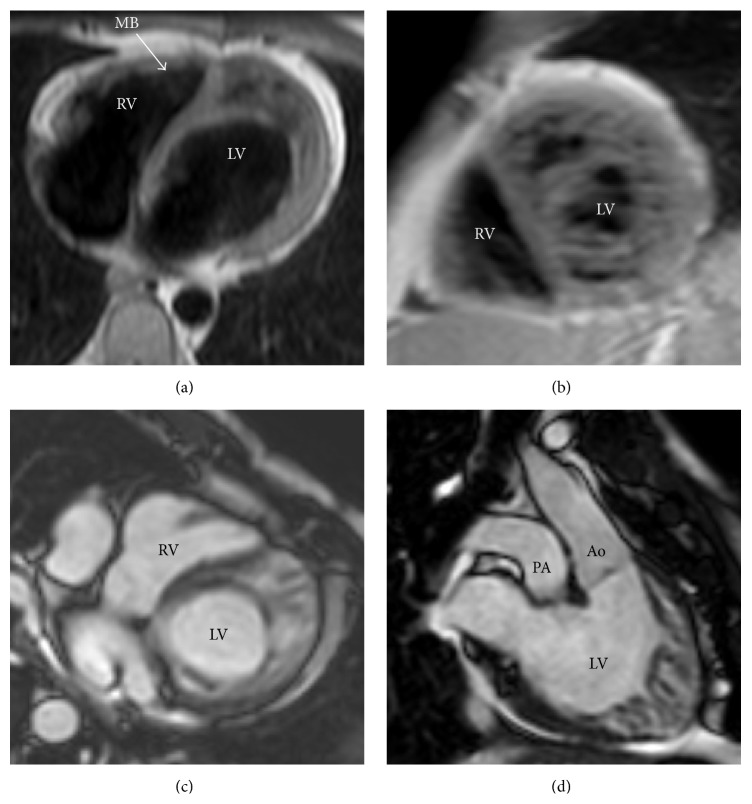
Cardiac MRI long axis (a) and short axis (b) black blood fast spin echo images of LV, with end-diastolic long axis ((c), (d)) cine balanced steady-state free precession acquisition, confirming the presence of the anatomic features of NC in the mid and apical segments. MB moderator band (arrow).

Coronary angiography showed a short main stem, originating from the right sinus of Valsalva, splitted into four branches, from right to left: an artery providing the right ventricle (RV) free wall (RCA), a dual left anterior descending (LAD) artery (type I variant of Spindola-Franco [7]), and finally a branch (Dx) supplying the anterolateral LV wall (Figures 1(a) and 1(b)). The lone circumflex artery (Cx) arose from the ascending aorta (Ao), just above the noncoronary posterior sinus, giving off branches to the inferolateral LV segments and the posterior interventricular septum (Figures 1(c) and 1(d)). No atherosclerotic disease was detected. Computed tomography (CT) (160-slice, Aquilion Premium, Toshiba Medical Systems, Otawara, Japan) angiography (Figure 2) confirmed the coronary anomaly excluding malignant, that is, interarterial, course but emphasized, after echocardiographic suspicion, a malposition of the great arteries, being aortic root leftward and anterior to a rightward and posterior pulmonary artery (PA). It also revealed ill-defined LV papillary muscles as well as the prominent trabeculations and deep intertrabecular recesses in communication with the LV cavity, typical of LVNC. For this reason, cardiac magnetic resonance (MRI) (1.5 Tesla, Philips Achieva; Philips Medical System, Best, Netherlands) completed the multimodal imaging assessment (Figure 3), demonstrating that the diagnostic criteria for LVNC [8, 9] were completely fulfilled. At first glance, the association of L-malposed Ao and LV hypertrabeculation led us to think about a borderline case of congenitally corrected transposition of the great arteries; nevertheless the normal RV morphology with a three-leaflet atrioventricular valve ruled out this diagnosis on behalf of the LVNC one, in a context of atrioventricular and ventriculoarterial concordance.

Because of frequent but not repetitive ventricular extra beats at the last in-hospital Holter dynamic ECG monitoring, he was discharged soon after loop recorder implantation. This device has never stored relevant arrhythmic events after 2-year follow-up.

## 3. Discussion

The embryonic myocardium is composed of a loose meshwork of interwoven fibers separated by deep recesses, which communicate with the LV cavity, allowing an increase in the myocardial surface area and the exchange diffusion from the cavity. From the 5th–8th week of embryogenesis, LV trabecular compaction occurs simultaneously with the invasion of the myocardium by the developing coronary vasculature coming from the epicardium: at the same time in fact the coronary circulation develops and intertrabecular recesses are transformed in capillaries [10]. The trabecular layer of the developing ventricular walls is known to compact from base to apex, from epicardium to endocardium, and from the septal to the lateral wall [1]. The underlying pathophysiologic mechanisms for lack of such compaction remain largely unsolved. The atrioventricular valves, papillary muscles, and chordae tendineae all form from portions of the ventricular walls as well. At the same embryonic period, the superior part of the interventricular septum develops from the aorticopulmonary septum, which is a spiral-shaped mass that also subdivides the truncus arteriosus into the PA and the ascending Ao: as a result of such spiral orientation of the septum, the Ao and PA twist around each other.

LVNC coexists with another cardiac anomaly approximately in 20% of cases [11]; sometimes it may be caused by an excessively high pressure exposure of the LV during intrauterine development. The American Heart Association statement on cardiomyopathies already classified LVNC within the genetic cardiomyopathies [12]: a mutation in the *α*-dystrobrevin gene was identified subsequently in patients with LVNC and associated with congenital heart diseases [13]. The clinical relevance of regional NC in the context of another cardiac malformation is still uncertain. About our patient, it is a suggestive hypothesis that such a complex anomaly originated just between the 5th and the 8th week of intrauterine life, when both myocardial compaction and coronary circulation development [10] as well as great arteries translocation occurred.

Diagnostic sensitivity has been enhanced by the introduction of specific morphologic criteria by high resolution echocardiography, CT angiography, and cardiac MRI. Echocardiography could pose inherent problems in assessing the LV apex [14] and lateral wall [15], known to be the most common NC areas. Conversely, the latter modalities may detect in more detail the degree of hypertrabeculation and the ratio between the trabecular and compact layers, as well as NC extension. Particularly cardiac MRI is accurate when diagnosing LVNC: a NC/C ratio of >2.3 in diastole distinguishes pathological NC, with values for sensitivity, specificity, and positive and negative predictions of 86%, 99%, and 75% and 99%, respectively [8]. Using MRI, Jacquier et al. [9] proposed a NC mass ≥20% of the total LV mass, as a further diagnostic criterion for the disease. In our case setting, the integrative nature of cardiac CT suggests an advantage of this imaging test over the others: it enabled the simultaneous noninvasive evaluation of the complex intracardiac pathology and ventricular function as well as the malposition of the great arteries and the coronary artery morphology.

The coincidence of LVNC, malposition of the great arteries, and such a complex coronary anomaly has never been described before. As this case illustrates, accurate diagnosis of a grown-up congenital heart disease could be very tricky, so much so that the role of multimodal imaging assessment is fundamental.

## References

[1] Sedmera D., Pexieder T., Vuillemin M., Thompson R. P., Anderson R. H. (2000). Developmental patterning of the myocardium. *Anatomical Record*.

[2] Jenni R., Oechslin E., Schneider J., Attenhofer Jost C., Kaufmann P. A. (2001). Echocardiographic and pathoanatomical characteristics of isolated left ventricular non-compaction: a step towards classification as a distinct cardiomyopathy. *Heart*.

[3] Weiford B. C., Subbarao V. D., Mulhern K. M. (2004). Noncompaction of the ventricular myocardium. *Circulation*.

[4] Almeida A. G., Pinto F. J. (2013). Myocardial disease. Non-compaction cardiomyopathy. *Heart*.

[5] Tatu-Chitoiu A., Bradisteanu S. (2008). A rare case of biventricular non-compaction associated with ventricular septal defect and descendent aortic stenosis in a young man. *European Journal of Echocardiography*.

[6] Oechslin E. N., Attenhofer Jost C. H., Rojas J. R., Kaufmann P. A., Jenni R. (2000). Long-term follow-up of 34 adults with isolated left ventricular noncompaction: a distinct cardiomyopathy with poor prognosis. *Journal of the American College of Cardiology*.

[7] Spindola-Franco H., Grose R., Solomon N. (1983). Dual left anterior descending coronary artery: angiographic description of important variants and surgical implications. *American Heart Journal*.

[8] Petersen S. E., Selvanayagam J. B., Francis J. M. (2005). Left ventricular non-compaction: insights from cardiovascular magnetic resonance imaging. *Journal of the American College of Cardiology*.

[9] Jacquier A., Thuny F., Jop B. (2010). Measurement of trabeculated left ventricular mass using cardiac magnetic resonance imaging in the diagnosis of left ventricular non-compaction. *European Heart Journal*.

[10] Engberding R., Bender F. (1984). Identification of a rare congenital anomaly of the myocardium by twodimensional echocardiography: persistence of isolated myocardial sinusoids. *The American Journal of Cardiology*.

[11] Espinola-Zavaleta N., Soto M. E., Castellanos L. M., Játiva-Chávez S., Keirns C. (2006). Non-compacted cardiomyopathy: clinical-echocardiographic study. *Cardiovascular Ultrasound*.

[12] Maron B. J., Towbin J. A., Thiene G. (2006). Contemporary definitions and classification of the cardiomyopathies: an American Heart Association Scientific Statement from the Council on Clinical Cardiology, Heart Failure and Transplantation Committee; Quality of Care and Outcomes Research and Functional Genomics and Translational Biology Interdisciplinary Working Groups; and Council on Epidemiology and Prevention. *Circulation*.

[13] Ichida F., Tsubata S., Bowles K. R. (2001). Novel gene mutations in patients with left ventricular noncompaction or Barth syndrome. *Circulation*.

[14] Moon J. C. C., Fisher N. G., McKenna W. J., Pennell D. J. (2004). Detection of apical hypertrophic cardiomyopathy by cardiovascular magnetic resonance in patients with non-diagnostic echocardiography. *Heart*.

[15] Alhabshan F., Smallhorn J. F., Golding F., Musewe N., Freedom R. M., Yoo S.-J. (2005). Extent of myocardial non-compaction: comparison between MRI and echocardiographic evaluation. *Pediatric Radiology*.

