# Corrigendum to “A Striking Coronary Artery Pattern in a Grown-Up Congenital Heart Disease Patient”

**DOI:** 10.1155/2016/8393704

**Published:** 2016-10-17

**Authors:** Fortunato Iacovelli, Martino Pepe, Gaetano Contegiacomo, Vito Alberotanza, Filippo Masi, Alessandro Santo Bortone, Stefano Favale

**Affiliations:** ^1^Interventional Cardiology Service, “Montevergine” Clinic, Via Mario Malzoni, 83013 Mercogliano, Italy; ^2^Division of Cardiology, Department of Advanced Biomedical Sciences, University of Naples “Federico II”, Via Sergio Pansini 5, 80131 Naples, Italy; ^3^Section of Cardiovascular Diseases, Department of Emergency and Organ Transplantation, University of Bari “Aldo Moro”, Piazza Giulio Cesare 11, 70124 Bari, Italy; ^4^Interventional Cardiology Service, “Santa Maria” Clinic, Via Antonio De Ferrariis 22, 70124 Bari, Italy; ^5^Section of Diagnostic Imaging, Interdisciplinary Department of Medicine, University of Bari “Aldo Moro”, Piazza Giulio Cesare 11, 70124 Bari, Italy; ^6^Section of Heart Surgery, Department of Emergency and Organ Transplantation, University of Bari “Aldo Moro”, Piazza Giulio Cesare 11, 70124 Bari, Italy

In the article titled “A Striking Coronary Artery Pattern in a Grown-Up Congenital Heart Disease Patient” [1], an acknowledgment should be added as follows.
